# Accumulation of Cytochrome *b*_558_ at the Plasma Membrane: Hallmark of Oxidative Stress in Phagocytic Cells

**DOI:** 10.3390/ijms23020767

**Published:** 2022-01-11

**Authors:** Stephenson B. Owusu, Sophie Dupré-Crochet, Tania Bizouarn, Chantal Houée-Levin, Laura Baciou

**Affiliations:** Institut de Chimie Physique UMR 8000, CNRS, Université Paris-Saclay, 91405 Orsay, France; stephenson-boakye.owusu@universite-paris-saclay.fr (S.B.O.); sophie.dupre@universite-paris-saclay.fr (S.D.-C.); tania.bizouarn@universite-paris-saclay.fr (T.B.)

**Keywords:** neutrophils, PLB-985 cells, NADPH oxidase, cytochrome *b*_558_, ROS, oxidative stress, ionizing radiation

## Abstract

Neutrophils play a very key role in the human immune defense against pathogenic infections. The predominant players in this role during the activation of neutrophils are the release of cytotoxic agents stored in the granules and secretory vesicles and the massive production of reactive oxygen species (ROS) initiated by the enzyme NADPH oxidase. In addition, in living organisms, cells are continuously exposed to endogenous (inflammations, elevated neutrophil presence in the vicinity) and exogenous ROS at low and moderate levels (travels by plane, radiotherapy, space irradiation, blood banking, etc.). To study these effects, we used ROS induced by gamma radiation from low (0.2 Gy) to high (25 Gy) dose levels on PLB-985 cells from a myeloid cell line differentiated to neutrophil-like cells that are considered a good alternative to neutrophils. We determined a much longer lifetime of PLB-985 cells than that of neutrophils, which, as expected, decreased by increasing the irradiation dose. In the absence of any secondary stimulus, a very low production of ROS is detected with no significant difference between irradiated and non-irradiated cells. However, in phagocytosing cells, irradiation doses above 2 Gy enhanced oxidative burst in PLB-985 cells. Whatever the irradiation dose, NADPH oxidase devoid of its cytosolic regulatory units is observed at the plasma membrane in irradiated PLB-985 cells. This result is different from that observed for irradiated neutrophils in which irradiation also induced a translocation of regulatory subunits suggesting that the signal transduction mechanism or pathway operate differently in both cells.

## 1. Introduction

Among the cells of innate immunity, neutrophils constitute the first line of defense against a broad spectrum of pathogens. Neutrophils, the most abundant in the blood circulation, exhibit nonspecific immunity by generating massive amounts of deleterious reactive oxygen species (ROS) into phagosomes in order to degrade internalized pathogens. This phenomenon is known as respiratory burst and leads to a massive endogenous source of ROS. The ROS production is initiated by the NADPH oxidase complex, an extremely efficient and highly controlled enzymatic system. NADPH oxidase is organized as a complex of membrane and cytosolic proteins in the active state. It is constituted of a membrane-bound flavocytochrome *b*_558_ (Cyt *b*_558_), which comprises two subunits, gp91^phox^ and p22^phox^, and four cytosolic components: p47^phox^, p67^phox^, p40^phox^ and Rac. Dysfunction in any of the NADPH oxidase components leads to chronic granulomatous disease (CGD), a condition that makes patients highly susceptible to infections.

Hyper-activation of this system or accumulation of neutrophils, as observed in acute inflammatory respiratory disease [1,2], generates exogenous sources of ROS, creating an oxidative stress situation. The actions of the elevated neutrophil presence and excessive production of ROS can assail neutrophils themselves and impair their functions, with a subsequent effect on neighboring cells or tissues. Alternatively, neutrophils can be exposed to other exogenous ROS sources such as ionized radiation that can range from low to high dose (<2 Gy to >25 Gy), which are equivalent to <0.68 µM O_2_^•^^−^ and 0.56 µM HO^•^ to 8.5 µM O_2_^•^^−^ and 7 µM OH^•^. Thus, levels of biological and chemical exogenous ROS to which neutrophils are subjected vary. The total exposure of living organisms to radiation causes a change in the composition of the blood related to the fact that hematopoietic tissue (bone marrow) is one of the most sensitive tissues to chronic or acute radiation exposure but circulating cells are also exposed. Several studies on the effect of high doses (>50 Gy) on white blood cells have been reported but with many contradictions regarding the effects of irradiation on neutrophil functions, such as superoxide production, bactericidal activity and chemotaxis response [3,4,5,6,7]. We have shown in a recent work that a critical parameter that should be taken into account when the effect of irradiation is studied is the number of surviving cells [8]. We investigated the relationship between the dose of irradiation at the cellular level and the activation of the NADPH oxidase complex at the molecular level. Our results indicated that radiation dose above 25 Gy acts as neutrophil stimuli, leading to a priming state of the NADPH oxidase complex and enhancement of respiratory burst due to NADPH oxidase activity [8]. This important observation could not be noted in a previous work since the purified components of NADPH oxidase, either individually or in the course of assembly, were subjected to exogenous ROS [9,10].

However, our daily lives involve exposures of circulating blood cells to lower levels of radiation (0–2 Gy), such as radiation workers, aircraft personnel and astronauts. Low dose irradiation (typically 2 Gy) is used in radiotherapy to kill cancer cells. The average total dose is usually 50 Gy but fractionated in ca. 2 Gy/irradiation depending on the cancer type. The side effects on patients, acute and latent, have been documented [11,12] and they are associated with the roles played by reactive oxygen species [13]. Aside from this, radiation workers, as well as flight passengers, pilots, stewards and astronauts who travel to space, receive some doses as well [14].

This has led us to investigate irradiation on phagocytic cells, from very low to higher doses. Alternatives for circulating blood cells (neutrophils) are PLB-985 cells, established in 1987 by Tucker et al. [15]. They constitute an advantageous substitute for several studies regarding neutrophil functions. They are human myeloid cell lines differentiated into neutrophil-like cells with similar morphological and functional properties to blood neutrophils. Like neutrophils, PLB-985 cells have been shown to undergo priming by TNF-α and GM-CSF, which enhanced NADPH oxidase activity [16,17]. They are frequently used as models of neutrophils and a leading model for cell motility and chemotaxis.

Thus, PLB-985 cells can be alternative for several studies regarding neutrophils. In this paper, our goal is to study the behavior of PLB-985 cells in the presence of an amount of ROS going from the nanomolar to the micromolar. We created ROS using gamma irradiation, which delivers quantitatively in solutions of similar ROS (OH and O_2_^•−^ free radicals, hydrogen peroxide) as those produced by neutrophils. We show that radiation-induced ROS leads to an expression of cytochrome *b*_558_, which was originally not present, in the plasma membrane of the PLB-985 (PLB) cells. This expression led to an increased response to ROS production when a secondary stimulus was introduced.

## 2. Results

### 2.1. Effect of Gamma Irradiation on PLB Cell Survival

**Figure 1:**
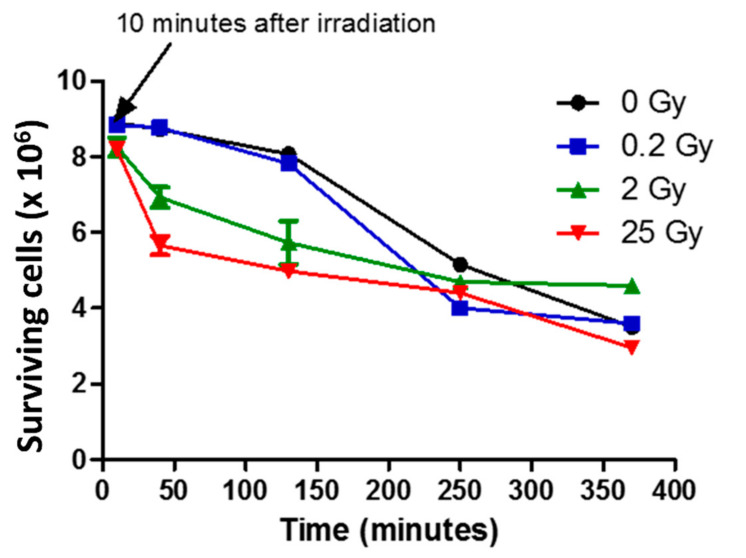
Effect of irradiation on PLB cell survival. Two milliliters each of differentiated PLB-985 cells (9 × 10^6^ cells/mL) were irradiated) at 0.2 Gy (0.2 Gy/min; square blue), 2 Gy (0.2 Gy/min; green triangle) and 25 Gy (25 Gy/min; red diamond). Non irradiated samples or sham (black circle) were kept at the same conditions as irradiated ones, except that they were not irradiated. Cells were then centrifuged and divided into 2 equal volumes (1 mL each, one of which was used to study cell death kinetics): 10 min after irradiation, the cells were counted under light microscope using trypan blue exclusion assay (*n* = 3). Subsequent counting was done at 40 min (*n* = 2), 130 min (2 h) (*n* = 2), 250 min (4 h) and 370 min (6 h). Surviving cells are the number of cells that had clear cytoplasm under light microscope. *n* = 1 for other counting times. The values are the average of the measurements ± SEM.

Surviving cells were counted under light microscopy. Trypan blue was used as a test for viable cells—cells with normal shape and clear cytoplasm under light microscope were counted as viable. Counting was done 10 min after irradiation, then after 40 min, 130 min, 250 min and 370 min. The survival curves of irradiated and non-irradiated PLB-985 cells are shown in Figure 1. Considering the survival curve of neutrophils (sham) determined in previous work [8], it was surprising to observe that PLB-985 cell deaths started in the course of time much longer than neutrophils and, in addition, with a different pattern. The course of time of irradiated PLB-985 cells appeared to be different depending on the irradiation dose. The cell death kinetics of 0.2 Gy irradiated cells followed a sigmoidal curve, similarly to the non-irradiated cells, with a relatively slight decrease of surviving cells during the first two hours followed by a drop to about 40% during the fourth hour after irradiation. The number of surviving cells irradiated with 2 Gy and 25 Gy followed a biphasic curve with a fast decay. It decayed strongly immediately after irradiation (within the first 40 min after irradiation) and afterwards slower to about 70% and 50% respectively. After this, the number of cells declined gradually. Interestingly ca. 40% of cells were still alive after 6 h.

### 2.2. ROS Production by Irradiated PLB-985 Cells

**Figure 2:**
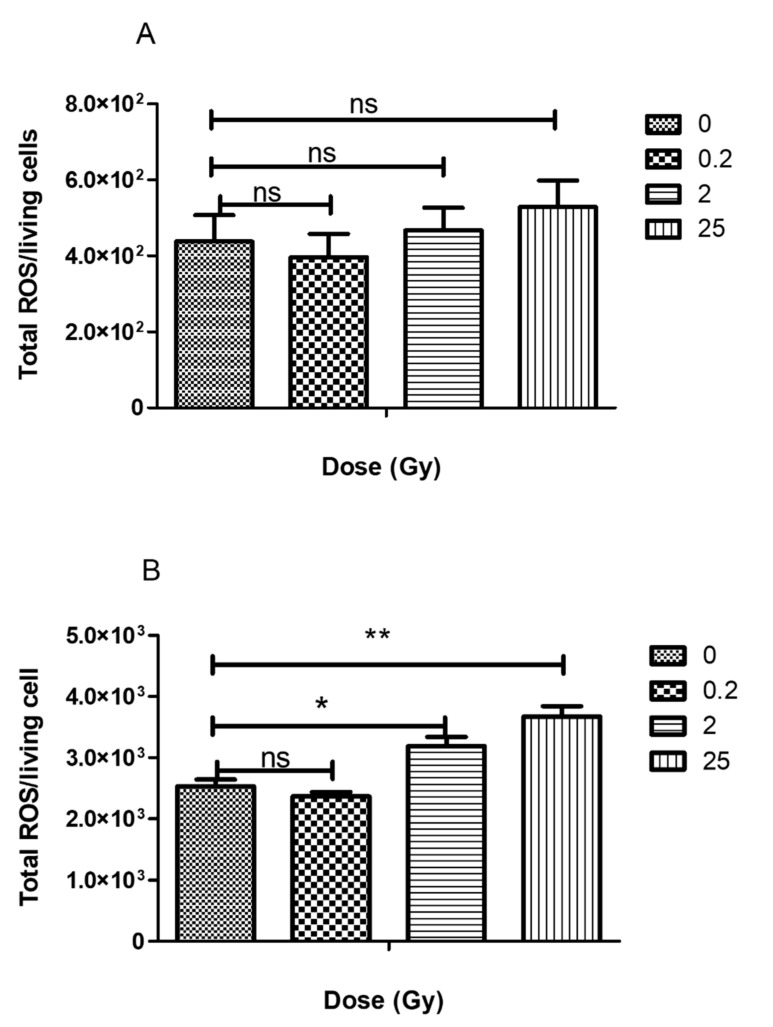
Irradiation effect on PLB-985 cells ROS production: (A) by unstimulated PLB-985 cells. ROS produced by unstimulated PLB-985 cells after irradiation were detected by chemiluminescence generated by the reaction of HRP and L-012 (see materials and methods). The area under chemiluminescence curve (Total ROS) was computed by the luminometer (B) By phagocytosing PLB-985 cells. PLB-985 were allowed to phagocyte zymosan opsonized with human serum for 2 h, and the ROS production was determined by the chemiluminescence generated by the reaction of HRP and luminol base L-012 (Figure S2). Data are represented as an average of 3 independent measurements ± SEM. ** *p* < 0.01; * *p* < 0.05; ns: non-significant.

The amount of ROS produced by irradiated and non-irradiated PLB-985 cells (in Hank’s buffer) was measured using chemiluminescence assay at 40 min after irradiation. The experiments were started using horseradish peroxidase (10 µg/mL) and L-012 probe (10 µM) in 96-well microplates without adding any activator and lasted for 2 h. The areas under the chemiluminescence curves (Figure S1) were normalized with the number of cells in each sample (Sham: 436,250 cells/mL; 0.2 Gy: 438,750 cells/mL; 2 Gy; 346,250 cells/mL; 25 Gy; 282,500 cells/mL). The results are shown in Figure 2A. Irradiated or non-irradiated neutrophil-like cells produced low level of ROS, with no significant statistical difference.

A similar experiment was performed after activating the cells (irradiated and non-irradiated) with opsonized zymosan (1 × 10^6^/mL). The area under the chemiluminescence plot generated (Figure S2) was normalized by the number of living cells (Figure 2B) used for the experiment (Sham: 436,250 cells/mL; 0.2 Gy: 438,750 cells/mL; 2 Gy; 346,250 cells/mL; 25 Gy; 282,500 cells/mL). The low dose (0.2 Gy-irradiation) did not lead to ROS production. In contrast, for higher doses, like neutrophils, phagocytosing PLB-985 cells displayed a significantly higher production of ROS than the unstimulated cells: 2 and 25 Gy-irradiation of PLB generated significant ROS production higher than the sham. The total ROS production per cell increased by about 30% for the 2 Gy and 60% for 25 Gy-irradiated cells compared to the sham. The phagocytic capacity of PLB-cells also showed the tendency to increase with irradiation after zymosan addition (Table 1).

Taken as a whole, our cell analyses show that low dose-irradiation of PLB-985 did not induce any detectable spontaneous ROS production but higher doses caused an increase of the oxidative burst, similar to what it was observed for 25 and 50 Gy-irradiated neutrophils.

### 2.3. Localization of NADPH Oxidase in Irradiated PLB-985 Cells

We aimed now to correlate the in cellulo enhanced oxidative burst to NADPH oxidase enzyme assembly at the membrane.

**Figure 3:**
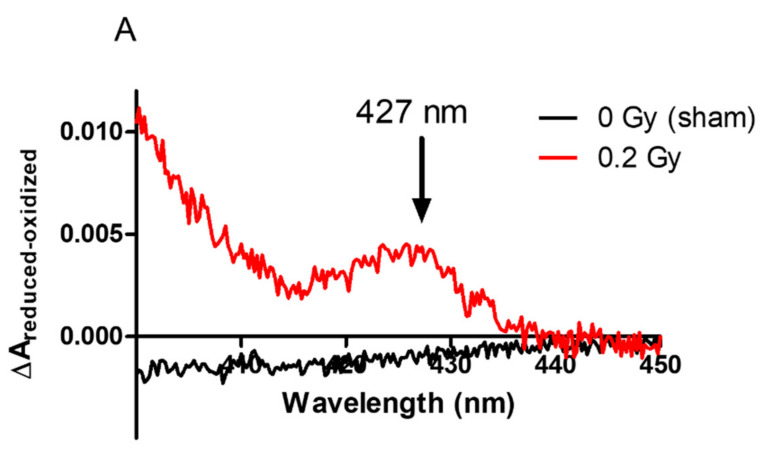

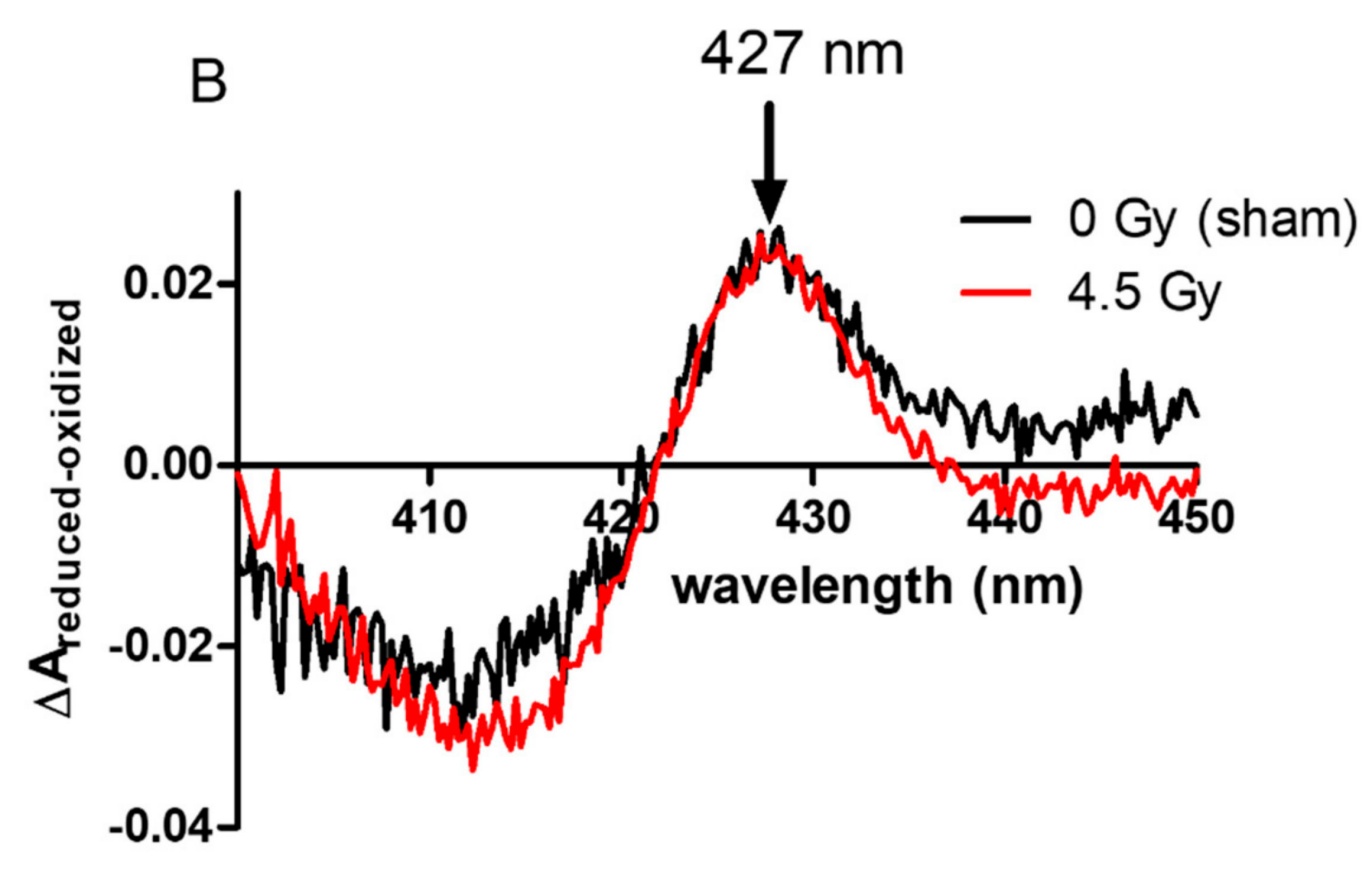
Dithionite reduced *minus* oxidized Soret band (427 nm) of membrane fractions purified (**A**) from irradiated (0.2 Gy) and non-irradiated PLB cells. (**B**) Irradiated neutrophil membrane fractions. The measured solution contained 100 µL of membrane fractions from surviving cells, 2 fold diluted and solubilized in 1% Dodecylmaltoside (DDM) in a quartz cuvette. The reference cuvette contained a similar solution. The reduced form was obtained by adding 1 mg of sodium dithionite to the sample cuvette after taking the oxidized spectrum. Observations were made with 2 or more independent preparations.

First, we determined the amount of cyt*b*_558_ at the plasma membrane from irradiated and non-irradiated PLB-985 cells (Figure 3A) and neutrophils (Figure 3B) by recording the dithionite-reduced *minus* oxidized difference absorption spectra. For membrane fractions isolated from the PLB-985 sham, no spectrum of cyt*b*_558_ was identified while the characteristic spectrum of cyt*b*_558_ was observed in the sham from neutrophils and all irradiated samples (PLB-985 or neutrophils). This result indicates that the amount of cyt*b*_558_ was undetectable in the sham membrane fraction from PLB-985 cells, negligible compared to the sham from neutrophils and that the irradiations boosted up the amount of cyt*b*_558_ in the plasma membrane.

**Figure 4:**
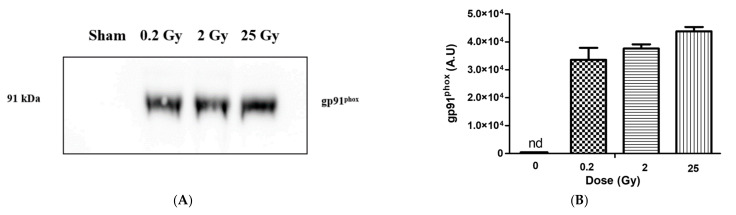
Presence of gp91phox in membrane fractions from irradiated and non-irradiated PLB cells. (**A**) Western blot of membrane fractions (gp91^phox^). Lane 1: 0 Gy (non-irradiated PLB-cells), lane 2, 3 and 4 represent PLB cells irradiated at 0.2 Gy, 2 Gy and 25 Gy, respectively. In each lane, 20 µg of total membrane proteins (determined from BCA assay) was deposited. The membrane fractions were heated for 5 min at 85 °C, and subject to 10% SDS-PAGE and immunoblotting. Each membrane was incubated with a primary antibody (dilution 1:1500 for gp91^phox^). (**B**) Band intensity of gp91^phox^ quantified by densitometry using ImageJ software (Java 1.8.0). nd represent non-determined. The Figure is a representation of 2 independent preparations.

We loaded the same amount of total membrane protein on SDS-PAGE and directed the antibody against gp91^phox^ (Nox2). The results are shown in Figure 4. We observed a very bright signal of gp91^phox^ in the membrane fractions from irradiated cells while, no signal was detected for membrane fractions purified from non-irradiated cells. The intensity of signals for the irradiated cells did not change amongst themselves within uncertainty. In Figure 4, by immunoblotting, we show that the increase arises from the presence of gp91^phox^ protein at the plasma membrane in irradiated cells.

**Figure 5:**
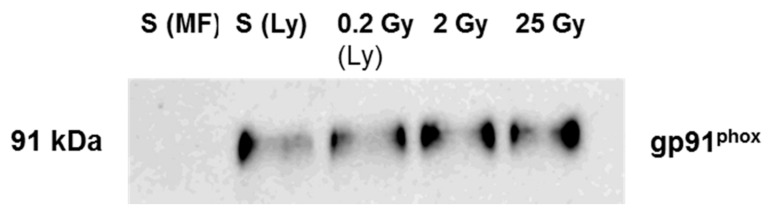
Gp91^phox^ detection in total lysate of irradiated and non-irradiated PLB cells_._ S(MF) represents the membrane fraction from sham (non-irradiated PLB-cells); S(Ly) represent the total cell lysate of sham; subsequent lanes (Ly) represents the total lysate of PLB cells irradiated at 0.2 Gy, 2 Gy and 25 Gy, respectively. In each lane, 20 µg of total membrane proteins (estimated from BCA assay) was deposited. The membrane fractions were heated for 5 min at 85 °C, and subject to 10% SDS-PAGE and immunoblotting. Each membrane was incubated with a primary antibody (dilution 1:1500 for gp91^phox^). MF: Membrane fractions, Ly: lysate.

To decipher if this increase is from the neo-expression of the proteins at the plasma membrane level or from recruitment from intracellular compartments, we performed a parallel experiment with the entire lysate of irradiated and non-irradiated PLB cells. We could see from Figure 5 that gp91^phox^ was detected in the sham (S(Ly)) cell lysate but not in the plasma membrane fractions purified from the sham (S(MF)) showing that in PLB cells, cyt*b*_558_ is localized principally at the level of intra-subcellular organelles. When cells are submitted to irradiation, cyt*b*_558_ is re-localized at the plasma membrane as observed already for a very low dose. In our previous work performed on irradiated neutrophils [8], we observed a pre-assembly of the NADPH oxidase complex. We therefore investigated if PLB cell irradiation similarly induced a partially pre-assembled NAPH oxidase. The hallmark of NADPH oxidase pre-assembly is the presence of the cytosolic component p47^phox^ at the membrane that we tested by immunoblot using an antibody against p47^phox^ (data not shown). Whatever the dose, no signal of p47^phox^ at the level of the plasma membrane could be detected indicating that, in contrast to irradiated neutrophils, NADPH oxidase was not pre-assembled by these irradiations including 25 Gy in irradiated PLB-985.

### 2.4. Activity of NADPH Oxidase in Irradiated PLB-985 Cells

Using BCA assays, we determined the total membrane protein concentrations in each sample. These value determinations are critical to accurately establish the specific activity of the enzyme. Based on the quantification data of the concentration of cyt*b*_558_, we determined the amount of cyt*b*_558_ per total membrane protein. Knowing the number of living cells that were broken, we could extrapolate to the average values of cyt*b*_558_ per cells. This calculation was done for each experiment and the average was performed for the cyt*b*_558_ per total membrane protein per cell. The results shown in Table 2 show that the concentration of total protein (using BCA) in membrane fractions isolated from irradiated living PLB-985 increased. The total membrane protein concentration slightly increased with increasing dose while the cyt*b*_558_ slightly decreased. However, when the number of cells that have been counted was taken into consideration, no more dose dependence was observable whatever the radiation dose. This calculation was not applied for sham since no cyt*b*_558_ was detected. An abrupt explosion of cyt*b*_558_ at the plasma membrane was observed with the first low dose irradiation.

**Figure 6:**
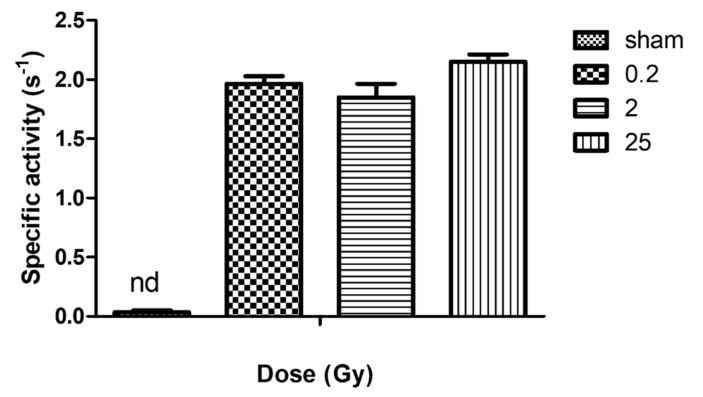
Specific activity of NADPH oxidase from irradiated and non-irradiated PLB-985 cells. Superoxide production was determined in vitro using membrane fractions purified from irradiated (4 nM cyt*b_558_*) surviving PLB-985 cells mixed with purified cytosolic fractions from each preparation (250 µg each) and incubated in activity buffer (PBS solution supplemented with 10 mM MgSO_4_) for 5 min in the presence of AA (40 μM). NADPH (200 μM) was added to initiate the reaction and 50 μM of cyt *c* to measure superoxide rate of production. The activity was calculated from the initial rate of reaction at 550 nm using an extinction coefficient of 21 M^−1^ cm^−1^. Specific activity indicates the amount of superoxide produced per second per mole of cyt*b*_558_ of NADPH oxidase. Data are the mean ± SEM of 3 independent experiments. nd represents non-determined for the non-irradiated sample.

These values were used to determine the specific activity of the NADPH oxidase (Figure 6).

These results confirm the qualitative analysis described above. We did not detect any NADPH oxidase activity in membrane fractions of non-irradiated PLB-985 cells. Conversely, in addition to similar expression or recruitment of cyt*b*_558_ to the plasma membrane, irradiation (irrespective of the dose) induced a similar superoxide production.

## 3. Discussion and Conclusions

PLB-985 cells are myeloid cell line differentiated to neutrophil phenotype presenting the advantage to be homogenous in density and phenotype based on the protocol used [18] and more reproducible than neutrophils from human blood. In this work, we explored from low (0.2 Gy) to high dose (25 Gy) levels of gamma irradiation on PLB-985, used as producers of known quantities of ROS. These cells are alternative model cells for several studies regarding neutrophils. It is of great interest to determine if their similarities to neutrophils allow studies in conditions of oxidative stress.

We showed that the decrease of survival PLB-985 cells for sham appeared only after 3 h. The 0.2 Gy-irradiated cells showed almost no changes compared to sham. This course of PLB-985 cell death is different from that of neutrophils, for which even the non-irradiated cells decline to about 85 percent within the first 30 min [8], indicating that PLB-985 cells survive much longer (in vitro in PBS). The cell behavior suggests that PLB-985 cells are more resistant to environment variations (maybe related to the absence of isolation procedures from donor blood pocket necessary for neutrophils). Thus, to this point of view, PLB-985 cells might be considered as convenient, stable neutrophil-like cells. For higher doses, as expected, the number of cells decreased by increasing the dose and this decrease occurred immediately after irradiation indicating that 2 Gy is a threshold for irradiation impact.

We did not detect cyt*b*_558_ at the plasma membrane in non-irradiated PLB-985 cells. It was shown that differentiated PLB-985 cells expressed NADPH oxidase components [17,18]. Antibody (7D5 monoclonal antibody) directed against the extracellular loop of gp91^phox^ in PLB-985 revealed that cyt*b*_558_ should be present at the plasma membrane [19]. However, the proportion of gp91^phox^ at the plasma membrane or in the intracellular compartment is unknown. This is partly due to difficulties in subcellular fractionation of PLB-985 cells [20]. In the non-irradiated cells, the amount of cyt*b*_558_ might be too low to be detected but interestingly, we could show that, if gp91^phox^ is not at the plasma membrane, it is present in the lysate probably at the intracellular organelles. Importantly this constitutes a good indication of cells in their resting state and permits to highlight the presence and the localization of cyt*b*_558_ in irradiated PLB-985 cells at the plasma membranes. This suggests that irradiation acts as a first stimulus triggering degranulation of the granules containing cyt*b*_558_. This is in line with an increased expression of cyt*b*_558_ or a recruitment of cyt*b*_558_ originally present in the intracellular compartments of PLB cells such as endosomes, secretory vesicles and specific granules. Pedruzzi et al. [17] have described degranulation of specific granules and secretory vesicles in PLB-985 cells although others suggested that PLB-985 cells lack secondary granules using three different cell differentiation media (dbcAMP, DMSO and DMF) [20]. These data are similar to our findings in our previous work done on neutrophils [8], except that no dose dependence was observed for unstimulated PLB-985 cells regarding the amount of cyt*b*_558_ and oxidase activity. This latter finding is consistent with the absence of cytosolic proteins at the level of the plasma membrane in irradiated cells but in contrast to our previous results for the same dose level in [8]. The mechanism of priming is complicated. Different agents with different concentrations of prime neutrophils through several signal transduction pathways [21,22]. TNF-α-mediated p47^phox^ phosphorylation is largely dependent on p38, while both ERK1/2 and p38 mobilize granules to the cell surface [23]. Therefore, it is likely that, in neutrophil-like PLB cells, low amounts of ROS activates the pathway of ERK1/2 leading to the sole cyt*b*_558_ recruitment at the plasma membrane. 

Finally, in the absence of any secondary stimuli, our data showed a very low production of superoxide with no significant difference between irradiated and non-irradiated cells. Only after phagocytosing activation, PLB-cells displayed the so-called oxidative burst corresponding to the activation of the NADPH oxidase enzyme. Cells submitted to 2 and 25 Gy exhibited a significant increase of the ROS production when exposed to a secondary stimulus (phagocytosis). This indicates that > 2 Gy-irradiation stimulated or prepared neutrophil-like cells to enhanced oxidative burst. This is in agreement with the long-standing model that the common pathway of priming is the enhancement of ROS production and is consistent with the results described in our previous work with neutrophils [8].

## 4. Materials and Methods

### 4.1. PLB Cell Culture and Irradiation

The human myeloid leukemia cell lines PLB-985 in our laboratory were a generous gift from Prof. Marie Jose Stasia, Grenoble. Differentiated PLB-985 cells were a generous gift from Prof. Oliver Nüsse. The protocol used to culture the cells was as follows: human myeloid leukemia cells PLB-985 were grown in RPMI medium containing L-glutamine (Gibco, Thermo Fisher Scientific, Illkirch, France) supplemented with 10% (*v*/*v*) FBS, 100 U/mL penicillin, 100 μg/mL streptomycin and 2.5 mg/L amphotericin B at 37 °C in a humidified atmosphere containing 5% CO_2_. The cells were then differentiated into neutrophil-like cells by adding 1.25% DMSO in exponentially growing conditions for 5 or 6 days.

𝛾-irradiation of the PLB-985 cells was carried out using the panoramic ^60^Co gamma-source IL60PL Cis-Bio International (France) at the ICP, University Paris-Saclay (Orsay, France). The dose rate was measured by Fricke dosimetry. The irradiation was done at room temperature. All details of doses and dose rates are gathered in Table S1 in the Supplementary Material.

In cell suspension, the effect of gamma radiation is mainly indirect due to the high percentage of water. The following species are formed: e_aq_^−^, HO^•^, H^•^, H_2_O_2_, H_2_, H_3_O^+^. In air atmosphere, only O_2_^•−^ and HO^•^ radicals with radiolytic yields 0.34 µmol/J and 0.28 µmol/J), are present during irradiation, in addition to H_2_O_2_ (0.07 µmol/J) thanks to the following reactions.

e_aq_^−^ + O_2__➝_ O_2_^•−^         k_1_ = 1.9 × 10^10^ M^−1^s^−1^ (1)

H^•^ + O_2__➝_ O_2_^•−^+ H^+^ ······k_2_ = 1.2 × 10^10^ M^−1^s^−1^ (2)

Two milliliters of suspended PLB cells (10^8^ cells/mL) were put into a 15 mL flat cap Falcon tube. One sample was kept as a sham (0 Gy). The other samples were irradiated at low and higher doses with the corresponding superoxide ions produced in the solution: 0.2 Gy (0.07 µM O_2_^•−^ and 0.06 µM HO^•^; 0.2 Gy/min dose rate), 2 Gy (0.68 µM O_2_^•−^ and 0.56 µM HO^•^; 0.2 Gy/min dose rate) and 25 Gy (8.5 µM O_2_^•−^ and 7 µM OH^•^; 25 Gy/min dose rate). Experiments were done in triplicates.

Prior to irradiation, 4 samples of PLB-985 cell suspension were made. Each sample contained 9 × 10^6^ cells/mL in a total volume of 2 mL. One sample was irradiated first with 2 Gy (dose rate 0.2 Gy/min). During this time, all the other samples were kept at room temperature. The other doses (0.2 Gy (dose rate 0.2 Gy/min), 25 Gy (dose rate 25 Gy/min)) were delivered successively, keeping a non-irradiated sample (sham). More than 98% of the counted cells in all samples for all preparations were viable after irradiation. Forty minutes after irradiation, the cells were centrifuged (400× *g* for 5 min), resuspended in lysis buffer (described in materials and methods) and broken by sonication. The number of living cells that were broken was considered. For these experiments, membrane fractions were isolated from 2 independent preparations. In another preparation, the entire lysate was used for further studies.

### 4.2. Zymosan Preparation

Zymosan (Z-4250, Sigma-Aldrich, Saint-Quentin Fallavier, France) were stained with Alexa 488 (Fisher, Illkirch, France): 100 µL of zymosan was incubated with 10 µL of Alexa 488 (5 µg/µL) in the presence of carbonate buffer at 37 °C for 1 h, washed three times and resuspended in PBS. Aliquots of 20 µL were stored at −80 °C. For ROS detection by luminometry, the zymosan was opsonized with complete human serum: 100 µL of zymosan was incubated with 100 µL of human serum on a wheel at 37 °C for 1 h, washed three times and resuspended in Hepes buffer or PBS. Aliquots of 20 µL were stored at −80 °C.

### 4.3. Phagocytosis Assay

Opsonized zymosan (10^6^) stained with Alexa 488 were added to the PLB-985 suspension (2 × 10^5^ cells). The suspension was mixed gently and put in an incubator at 37 °C for 30 min. The cells were transferred into cytometry tubes containing 600 µL of PBS, and phagocytosis was monitored using CyFlow LL flow cytometer (Sysmex Partec, Norderstedt, Germany) by recording the fluorescence intensity. Parallel negative controls were also prepared. To stop the phagocytosis, the cytometry tubes were transferred in a cold bath. To measure the actual phagocytic capacity of neutrophils, the fluorescence was quenched with trypan blue. Results were then analyzed using FlowJo software, version 10.

### 4.4. In Cellullo ROS Detections

ROS production during phagocytosis was detected using a chemiluminescence probe. In brief, cells were resuspended in Hank’s buffer, 10 µg/mL horseradish peroxidase (Sigma-Aldrich, Saint-Quentin Fallavier, France) and 10 µM luminol-based L-012 (Sigma Aldrich, Saint Quentin Fallavier, France) were added to the cells before the addition of zymosan opsonized with human serum and the chemiluminescence was measured for 2 h using Hybrid Multi-Mode Microplate Reader, Synergy^TM^H1 (BioTek Instruments, Winoski, VT, USA) luminometer.

### 4.5. Purification of Plasma Membrane from PLB-985 Cells 

Each subset of PLB-985 cells (0, 0.2, 2 and 25 Gy) dedicated to the purification of plasma membrane fractions were centrifuged 400× *g* for 8 min, cell viability (>98%) was verified by trypan blue exclusion assay to remove dead cells and resuspended in lysis buffer (20 mM phosphate buffer, pH 7.4, 340 mM sucrose, 7 mM MgSO_4_, 0.2 mM leupeptin and 1 mM PMSF). The cells were broken by sonication, followed by centrifugation for 15 min, at 10,000× *g* at 5 °C. This step leads to a pellet containing granules, mitochondria, unbroken cells and other organelles. The PLB-985 membrane fractions were then separated from the cytosolic ones by further centrifugation for 1.5 h at 200,000× *g*, 5 °C. The membrane fraction was re-solubilized in the lysis buffer by a brief sonication and stored at −80 °C in aliquots of 50 μL for further use. Aliquots of the cytosolic fraction (supernatant) were also made and stored at −80 °C. The protein concentrations were determined using Pierce BCA assays. The cyt*b*_558_ concentration was quantified from the difference absorption spectrum (reduced *minus* oxidized at wavelengths 411 nm, 427 nm and 558 nm) using the extinction coefficient of 200 mM^−^^1^·cm^−^^1^ at the Soret band (427 *minus* 411 nm). The difference spectra of reduced (by dithionite) *minus* oxidized cyt*b*_558_ was recorded from each neutrophil membrane preparation. The membrane fraction (100 µL) was solubilized in 1% dodecyl maltoside (DDM) to decrease sample turbidity and to prevent light scattering.

For Western blots analysis, the SDS-PAGE was carried out using 8% to 12% polyacrylamide resolving gel containing 0.1% SDS and 5% stacking gel. The resolved proteins were electro-transferred from acrylamide gel to a nitrocellulose membrane sheets (GE Healthcare) as described in [24]. The assembly was placed in a 20 mM CAPS buffer pH 11.0 containing 10% methanol. The transfer was carried out cold and stirred for 1 h at 150 V; 250 mA. The nitrocellulose membrane was incubated for 1 h in a 2.5% solution of Bovine Serum Albumin prepared in 20 mL of 1xTBST containing 0.075% Tween-20 on an orbital shaker at room temperature. The sheets were then incubated overnight at 4 °C with specific monoclonal or polyclonal antibodies: anti-gp91 (anti-gp91^phox^; 54.1; mouse monoclonal, dilution 1:1500; Santa Cruz Biotechnology, Heidelberg, Germany), anti-p22 (44.1; mouse monoclonal; dilution 1:1500; Santa Cruz); anti-p40 (D-8; mouse monoclonal; Santa Cruz), anti-p67 (rabbit polyclonal; dilution 1:1000; Sigma Aldrich); anti-p47 (rabbit polyclonal; dilution 1:1500); anti-Rac2 (C-11; rabbit polyclonal; dilution 1:1500), anti-Rac1 (ARCO3; Cytoskeleton); anti-p47-P-ser328 and anti-p47-P-ser345 (rabbit) were a kind gift from Dr Jamel El Benna). The immune complex was detected with either goat anti-rabbit (dilution 1:15,000; Santa Cruz) or goat anti-mouse (dilution 1:15,000; Santa Cruz) secondary antibodies conjugate to peroxidase. The bound peroxidase activity was detected by an imaging system (PXi, Syngene, Ozyme, Montigny-le Bretonneux, France) using ECL reagents (Prime Amersham, Fisher Scientific, Illkirch, France).

### 4.6. The In Vitro NADPH Oxidase Activity

Superoxide production by NADPH oxidase was determined in vitro using membrane fractions purified from irradiated (4 nM cyt*b_558_*) and non-irradiated (non-determined) surviving PLB-985 cells mixed with purified cytosolic fractions from each preparation (250 µg each) and incubated in activity buffer (PBS solution supplemented with 10 mM MgSO_4_) for 5 min in the presence of AA (40 μM). NADPH (200 μM) was added to initiate the reaction and 50 μM of cyt *c* to measure the superoxide rate of production. The activity was calculated from the initial rate of reaction at 550 nm using an extinction coefficient of 21 M ^−1^cm^−1^. Specific activity indicates the amount of superoxide produced per second per mole of cyt*b*_558_ of NADPH oxidase.

### 4.7. Statistical Analysis

All data are presented as mean ± SEM. Significance was determined using Student’s *t* test or Repeated Measures ANOVA. A *p*-value of ≤0.05 was considered statistically significant.

## Figures and Tables

**Table 1:** Phagocytic activity of PLB cells Zymosan (10^6^/mL) stained with Alexa 488 were added to the PLB suspension (50 µL each). The suspension was mixed gently and phagocytosis assay was performed at 37 °C for 30 min. The phagocytic activity was expressed as (% phagocytic cells containing ≥ 1 zymosan) × (mean fluorescence of the phagocytic cells). Results are presented as the mean ± SEM of two independent experiments.

Dose (Gy)	Phagocytic Activity
Sham	2011 ± 395
0.2	2123 ± 128
2	3082 ± 452
25	2467± 334

**Table 2:** Determination of cyt*b*_558_ amount in plasma membranes isolated from irradiated and non-irradiated PLB-985 cells Cyt*b*_558_ contents in the plasma membrane were estimated from the difference absorption spectra and the total membrane proteins (tMP) were determined using BCA assay. Data are mean ± SEM of two independent experiments. Nd: not determined.

Dose (Gy)	Total Membrane Proteins (mg/mL)	[cyt*b*558] (µg/mL)	Total Number of Cells Broken (×10^6^)	mg cyt*b*_558_/mg Total Membrane Proteins/Cells (×10^−11^)
0	4.01 ± 0.04	nd	17.5 ± 0.1	nd
0.2	4.90 ± 0.01	8.0 ± 1.0	17.6 ± 0.1	9.27 ± 0.79
2	5.09 ± 0.13	7.0 ± 0.0	13.9 ± 0.6	9.89 ± 0.82
25	5.2 ± 0.12	5.0 ± 0.0	11.3 ± 0.5	8.51 ± 0.86

## Supplementary Materials

The following supporting information can be downloaded at: https://www.mdpi.com/article/10.3390/ijms23020767/s1.
