# Supplementary material for: Accumulation of Cytochrome b558 at the Plasma Membrane: Hallmark of Oxidative Stress in Phagocytic Cells
Source: Int J Mol Sci. 2022 Jan 11;23(2):767. doi: 10.3390/ijms23020767 (PMC8775928; doi:10.3390/ijms23020767)
Supplement: Supplementary file 1 [file ijms-23-00767-s001.zip › ijms-1519776-supplementary.pdf]

Supplementary material

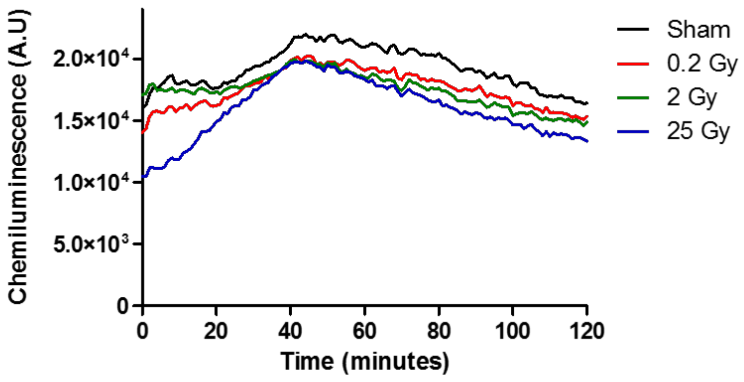

Figure S1. Chemiluminescence plot generated by unstimulated PLB-985 cells. ROS produced by PLB-985 cells after irradiations were detected using chemiluminescence during 2 hours by the reaction of HRP and luminol base L-012

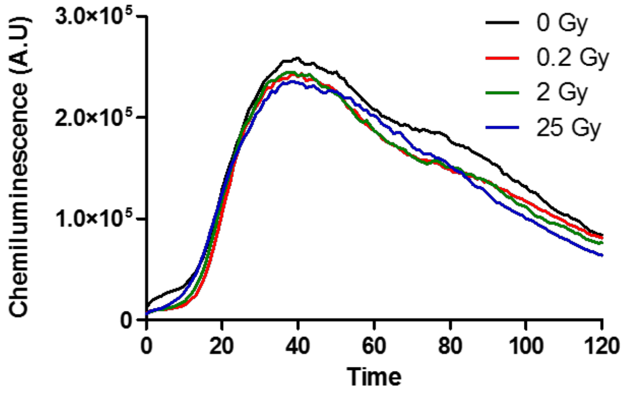

Figure S2. Chemiluminescence plot generated by phagocytosing PLB-985 cells. ROS produced by PLB-985 cells after irradiations were detected using chemiluminescence during 2 hours by the reaction of HRP and luminol base L-012

Table S1. Doses, dose rates and the total amount of OH radicals generated

**Dose rate (Gy/min)**	**Irradiation time (min)**	**Dose received (Gy)**	**Total amount of OH radicals**
0.2	1	0.2	56 nM
0.2	10	2	5.6 µM
21.5	1.2	25.8	72 µM
